# Biomechanical Tests on Long-Bone Elliptical Medullary-Canal Endoprostheses for Limb Salvage in Dogs

**DOI:** 10.3390/ani12213021

**Published:** 2022-11-03

**Authors:** Rosa Mendaza-DeCal, Yolanda Ballesteros, Salvador Peso-Fernandez, Juan Carlos del Real-Romero, Jesus Rodriguez-Quiros

**Affiliations:** 1 Animal Medicine and Surgery Department, Veterinary Faculty, Complutense University of Madrid, 28040 Madrid, Spain; 2 Abax Innovation Technologies, 28691 Villanueva de la Cañada, Spain; 3 Mechanical Engineering Department, Institute for Research in Technology, Universidad Pontificia Comillas, 28015 Madrid, Spain

**Keywords:** biomechanical test, torsional test, FDM, PEEK, exo-endoprosthesis, ex vivo, radius, canine

## Abstract

**Simple Summary:**

Currently, more owners look for offering a better quality of life to their pets. In fact, the complete limb amputation seems to be the last option considered by pet owners in surgeries to save their pets’ lives. Although this field is under development in veterinary medicine, we believe that 3D-printed implants for this market sector will improve the advancement in its research by reducing production costs. This would allow the pet owners to select this solution without large expenses, allowing at the same time, advances in this field. For this purpose, mechanical tests have been carried out on implants printed in a high-performance plastic that resembles the resistance of metals—that are traditionally used in veterinary surgery—and the properties of dogs’ bones as well. The results obtained have confirmed that the implants could withstand the dog weight in its different gaits, although further comparative studies on the effect of rotation forces applied during the animal’s change of direction (evaluated at different paces) are required to confirm their suitability.

**Abstract:**

Exo-endoprosthesis is a limb salvage procedure poorly described for animals, as only expensive metal devices have been used so far. Currently, additive manufacturing (AM) can make this type of implant affordable by exploring a wide new range of materials. However, safety factors should be considered and could be related to kinetic and kinematic studies of canine natural gaits. The suitability of a novel inner part of an exo-endoprosthesis manufactured by fuse deposition modeling (FDM) was assessed for long canine bones with an elliptical medullary canal. Polyether ether ketone (PEEK) was the material used as an alternative to metal for veterinary traumatology. Poisson’s ratio of 3D-printed PEEK material and ex vivo mechanical tests of the customized endoprosthesis were performed for the evaluation. The customized endoprostheses had promising outcomes for the radii of 20 kg dogs. Quasistatic mechanical tests of bone-inserted endoprostheses—pure compression tests—reached a maximum force of 1045.0 ± 78.0 N. In fatigue tests, the samples reached 500,000 cycles without failure or detriment to their quasistatic results. These outcomes surpass the natural weight-bearing of dogs, even during a galloping pace. Furthermore, torque tests with different adhesives were performed to obtain reference data for future assessments comparing with natural dog movements.

## 1. Introduction

Natural quadrupedal locomotion is always the objective in veterinary traumatology and orthopedics. Therefore, it would be natural to maintain this goal even in those cases where amputation is proposed. Most performed limb amputations are total limb amputations and associated with the aggravation of an animal’s concomitant orthopedic diseases [1,2,3,4,5,6], and the recent refusal of pet owners to perform them [1,2,6]. Consequently, some alternatives are being tried following actual treatment options from human medicine: socket prosthesis and exo-endoprosthesis [7,8,9,10,11,12,13,14].

In veterinary medicine, there are some examples of socket prostheses with dissimilar outcomes [7,8,9]. On the other hand, fewer cases of exo-endoprosthesis have been described in the literature with quite interesting results [11,12,13,14]. Both types of prosthesis are fabricated with commonly used materials in human and veterinary medicine, so these studies are focused on clinical reports instead of assessing the devices with in vitro or ex vivo mechanical tests. Only one research paper details mechanical tests for exo-endoprosthesis devices which are manufactured by additive manufacturing (AM) [10]. This device is made of metal, and neither bone nor animal biomechanics were considered in its mechanical tests because metal materials are widely used for orthopedic implants.

In veterinary medicine, pet owners pay the costs of these devices. Sometimes, owner’s economic limitations make it impossible to choose these solutions; therefore, innovation and evolution in this field can be difficult. DeVasConCellos et al. [10] used AM for metal-made endoprosthesis due to the possibility of patient-specific fabrication. However, they did not specify the economic savings of this technology in comparison with traditional manufacture [15]. VasConCellos’ devices [10] were manufactured by Direct Metal Laser Sintering (DMLS) technology, which is much more expensive than Fused Filament Fabrication (FFF) technology [16]—around 85% of savings for printing a part using FFF. In addition, FFF is mainly a plastic manufacturing technology, thus a wide variety of materials can be chosen. Considering this variety of options, they have already been used as an alternative to metals [17,18]. An interesting alternative as a polymer is polyether ether ketone (PEEK) [19]. It is demonstrated that 3D-printing reduces mechanical properties of polymers, and PEEK is no exception [20]. Its mechanical properties, even if printed models had 100% infill, were inferior to those of injection-molded PEEK due to the porosity that remains after the 3D-printing process [21]. Despite this reduction, the Young’s modulus of 3D-printed PEEK is closer to cortical dog bone ranges than metallic materials [22,23,24]. When porosity was incorporated into PEEK implants, it was identified as an effective approach to improve PEEK osseointegration [22,25].

Over the years, stress-shielding has been proven to be a significant postoperative complication of metal orthopedic devices [26,27,28,29]. This complication is a mechanical phenomenon where several factors are involved such as size, shape, and density of bone and material, shape, and implant size. All these factors are related to the elastic properties of the implant and bone which can be summarized in Young’s modulus and tensile strength. Meanwhile, surgical metals are more than 10 times stiffer than canine bone, PEEK is only 0.2-fold stiffer. A ratio of the PEEK Young’s modulus to that of bone of less than one indicates greater PEEK flexibility. It has been demonstrated that lower stiffness of the implant improves bone ingrowth and a denser medullary bone in the bone–implant interface [28,29]. In the veterinary field, this stress-shielding has been shown especially in the radius of light-weight patients with dynamic compression plates [30,31,32]. These findings should lead the veterinary community to search for other more flexible materials without losing weight-bearing and fatigue strength properties, so polymers could be an interesting alternative.

For assuring that polymer devices can effectively withstand the activity and weight of animals, using natural mechanical forces borne by their limbs is recommended as a reference for working load to determine a factor of safety (FoS). There are multiple studies concerning the natural quadrupedal biomechanics of dogs [33], and less for cats [34]. These studies record kinematic and kinetic properties, such as joint angles and ground reaction forces, during the different paces of dogs and cats [33,34,35,36]. Maximum and minimum joint angles of the forelimb are recorded at different velocities. During walking, the carpus joint varies from 128° to 239°, dorsally measured in the sagittal plane [37]. During trotting, these joint angles go from 63° to 215° [38]. During maximal movement initiation, the carpus joint angles go from 48° to 191° [39]. However, bone angles regarding the ground are not typically assessed [33,40].

During these movements, forelimbs withstand different body weights (BW) according to type of pace and moment of stance [33]. During walking, each canine thoracic limb bears around 0.61-fold BW [33], whereas each feline thoracic limb withstands nearly 0.58-fold BW [34]. In a trotting pace, each thoracic limb bears around 1.3-fold BW [33], while if dogs are in a steady gallop, each thoracic limb bears 2.1 times of BW. These weight-bearing data were normalized to animal body weight using the BW equation of Krotscheck et al. [41]. Consequently, instead of just expecting device failure [10,42,43], these “natural” values could be considered as a reference for ex vivo mechanical tests of new implants. Hence, the FoS could be readjusted for evaluating new, non-metallic materials as was previously described for canine bones with cylindrical medullary canals in Mendaza et al. [20].

Torsional loads are described for fractures of different canine long bones at high or slow speeds by Sammarco et al. [44]. Instead, studies about torsional loads have not been described during different paces or changes of direction at different velocities. Torsional loads during different paces would be a reference for assessment of the viability of limb salvage implants and anchorage union between the internal implant and the external attachment.

The aim of this study was an ex vivo assessment of the biomechanical behavior of 3D-printed PEEK for an intraosseous transcutaneous implant for bones with an elliptical medullary canal. Implants were inserted into canine radii which received static (pure compression and torsional loads) and dynamic loads in a vertical fashion, imitating general canine carpus angulation, contrary to what has recently been published for canine tibiae such as bones with a cylindrical medullary canal [20].

## 2. Materials and Methods

### 2.1. Endoprosthesis Design

**Figure 1:**
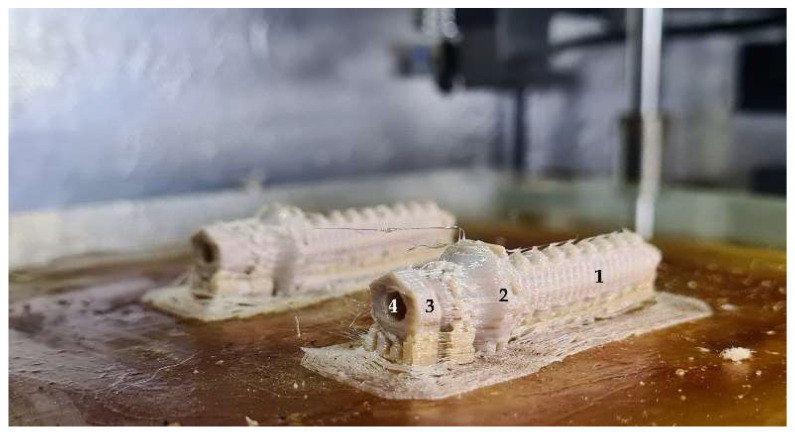
Two elliptical PEEK parts of the endoprosthesis just out of the 3D printer. (1) stem-section, (2) “umbrella”, (3) base, and (4) inner cavity where the surgical metal threaded rod is screwed.

The endoprosthesis device was designed to fit any patient’s medullary canal and patented by Mendaza et al. [45] (Figure 1). This device was composed of two main parts: (1) the PEEK part which would be in contact with the bone tissue; and (2) the surgical metal threaded rod which would be attached to an exoprosthesis. The PEEK part was comprised of a base, “umbrella”, neck, and stem. The assessed threaded rod was made of AISI 316 austenitic stainless steel.

The PEEK part of the endoprosthesis was designed using a computer-aided design program (SolidWorks, SolidWorks Corp., Waltham, MA, USA). Medullary cavity sizes of radii were obtained as a reference for stem external diameters and transversal shape of the endoprosthesis. Due to the elliptical shape of radii medullary canals and their lack of correlation between cranio–caudal and medio–lateral dimensions [46], both bones and endoprostheses were classified by cranio–caudal diameter of the radii. Radii with an average cranio–caudal medullary canal diameter (also short diameter) of 6.46 ± 0.29 mm were selected. The radius medullary canal was measured by a metric digital caliper. Stem external diameters were 0.22 mm larger than radii medullary canals for the short diameter; and 0.07 mm smaller than the long diameter of the radii. The inner cavity diameter of the PEEK part was 4 mm, as was the threaded rod diameter. This cavity was slightly reduced at the stem area for applying an extra radial compression to the bone, such as described by Mendaza et al. [47].

### 2.2. 3D Printing Filaments

Radial endoprostheses were printed in PEEK (3D4Makers, Amsterdam, The Netherlands). Filaments were stored in a special zip-lock multi-layered bag with an EVOH barrier film and silica desiccant sachets provided by the filament supplier. Before the printing process, spools were dried at 150 °C for three hours in a dry heat oven, following the other authors’ recommended protocols [23].

### 2.3. 3D Printing Parameters and Direction

PEEK samples and endoprostheses were printed by FunMat HT (INTAMSYS, Shanghai, China) based on FFF technology, with a build volume of 260 × 260 × 260 mm (x, y, z). The printer had a hardened-steel nozzle of 0.4 mm diameter. This printer reached a nozzle temperature up to 450 °C, bed temperature up to 160 °C, and chamber temperature up to 90 °C. Before printing, 3D models were sliced using Simplify3D^®^ software (version 4.1.2., Simplify3D, Cincinnati, OH, USA).

**Figure 2:**
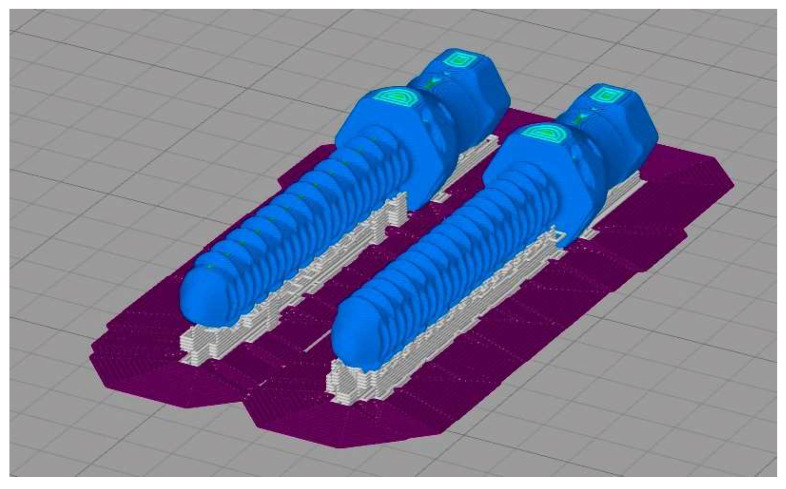
Printing simulation of radii endoprostheses on Simplify 3D slicer.

All models were horizontally oriented with respect to the printing bed [47]. The endoprosthesis vertical axis, and plane of the large diameter of endoprosthesis were parallel and perpendicularly oriented to printing bed, respectively (Figure 2). Printing orientation was important considering the main mechanical forces borne inside the medullary canal of the bone. Temperature parameters were nozzle temperature (410 °C), bed temperature (130 °C), and chamber temperature (90 °C). The fabrication code (.factory) was divided into three processes whose difference was in cooling percentage for different heights. The rest of the printing parameters are shown in Table 1. Cooling percentages were 20, 30, and 20 for processes 1, 2, and 3, respectively.

Immediately before each printing, a liquid fixative (Dimafix, DIMA 3D, Valladolid, Spain) for 3D-printing was applied to the cold print bed for better adhesion of the material during printing. Before starting any PEEK printing, a preheating of the chamber and build bed temperatures were set and allowed to stabilize for at least 30 min.

### 2.4. Preparation of the Endoprosthesis–Radius Construct

**Figure 3:**
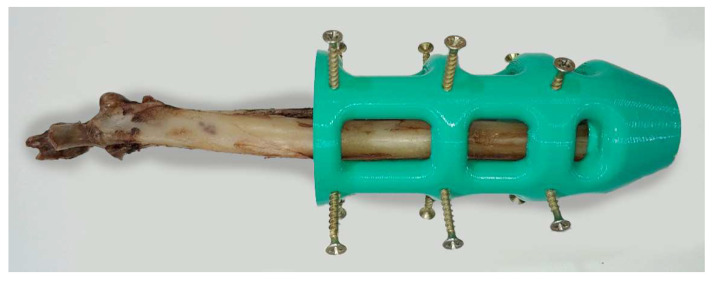
Concentric drill guide. It was self-designed, and 3D printed in PLA.

The endoprosthesis–radius interface was assessed by mechanical tests [47]. Ex vivo fresh radii were used to insert PEEK devices. Twenty-three radii used for this study came from canine specimens (20.85 ± 1.25 kg BW) which were euthanized for reasons unrelated to this study. Soft tissue was removed from the radii, and bones were immediately stored inside a vacuum bag in a freezer at −18 °C until to endoprosthesis insertion. Bones were perpendicularly cut to the bone longitudinal axis above the distal epiphysis and the short diameter was drilled with a 6 mm of diameter bit. Two PLA 3D-printed self-designed surgical guides were used for making perpendicular cuts and aligned drilling (Figure 3). Endoprosthesis insertion was made by softly hammer blowing. Later, the stainless steel 316 threaded rod with a 4 mm of diameter was gently inserted using a locknut. Furthermore, 30 mm length was left outside the PEEK part.

Once the endoprostheses were inserted, the radii were attached with a two-component epoxy resin (Resoltech 1050/1058S, Eguilles, France) in aluminum holders at 90° to the ground. This angle was chosen to ensure the repeatability of the tests, although the maximum weight-bearing is 95° [33]. Bone specimens were covered with gauze soaked in phosphate buffer solution for compression and fatigue tests to maintain the pH and humidity degree.

### 2.5. Mechanical Testing

#### 2.5.1. Mechanical Characterization of 3D-Printed PEEK

**Figure 4:**
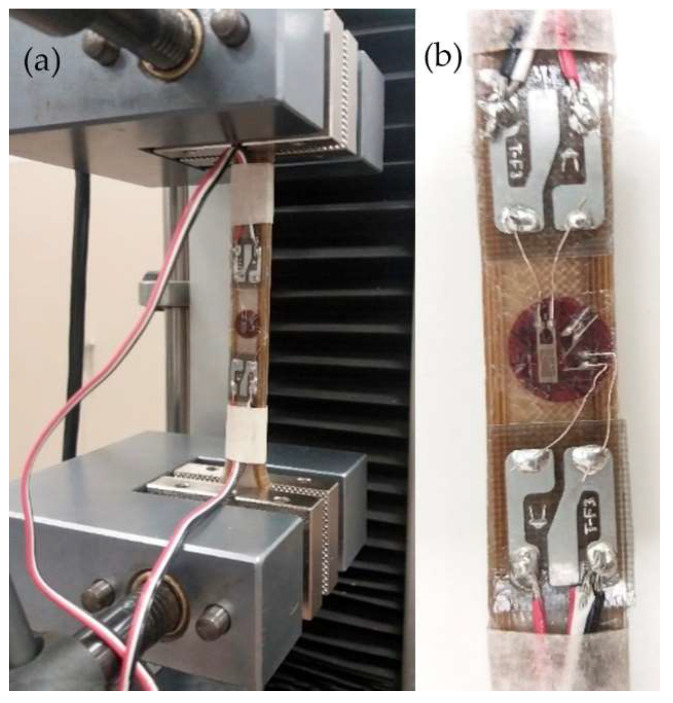
(**a**) Poisson’s ratio test using bidirectional strain gauges on a dog bone shaped specimen; (**b**) strain gauge in detail.

Mechanical properties of the 3D-printed PEEK (Young’s modulus, tensile strength, and bending strength) had already been determined in a previous study [20]. To determine de Poisson’s ratio of the 3D-printed PEEK, an increasing load was applied with a Universal Testing Machine IBTH/500 (SAE Ibertest, Madrid, Spain) (Figure 4). Deformations (εx and εz) were measured with bidirectional strain gauges (Kyowa Electronic Instruments Co, Ltd., Tokyo, Japan). Deformation data were recorded with a MX840B DAQ system (Hottinger Brüel & Kjaer GmbH, Darmstadt, Germany) (Figure 4). Three dog bone-shaped samples of 3D-PEEK were used for this test.

The results of the Poisson’s ratio were compared with those of canine radius, 316L stainless steel (316L-SS), and titanium alloys (Ti alloys) obtained from the literature (Table 2). Young’s modulus and the tensile strength of 3D-printed PEEK were recollected from a previous study.

#### 2.5.2. Quasi-Static Mechanical Testing of the Endoprosthesis–Radius Construct

**Figure 5:**
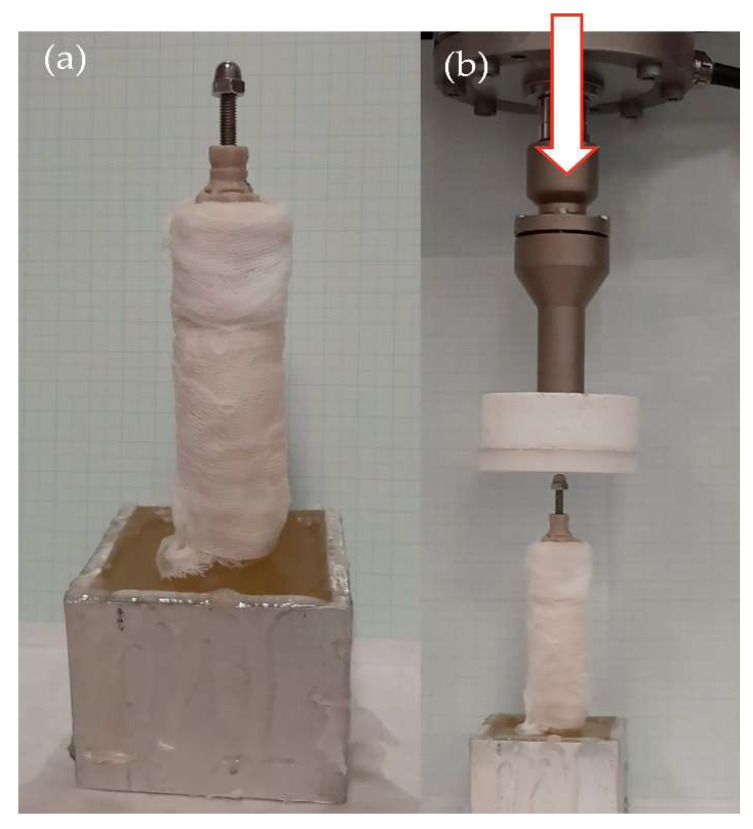
(**a**) Prepared endoprosthesis–radius construct for mechanical testing cured in an aluminium holder, perpendicular with respect to the testing table; (**b**) endoprosthesis–radius construct place at fatigue machine.

To obtain a more realistic result for the mechanical behavior of the endoprosthesis–radius construct, constructs were prepared as described in Materials and Methods for compression (90°) testing. Eight constructs were evaluated using a Universal Testing Machine Elib 20W (SAE Ibertest, Madrid, Spain) with a load cell of 2 kN at a crosshead speed of 500 mm/min (Figure 5).

**Figure 6:**
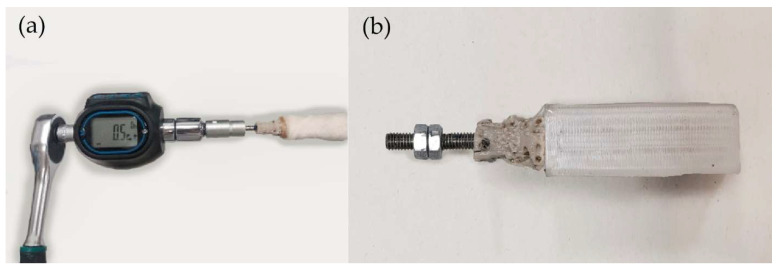
Specimen preparation for the torsion test. (**a**) Torque force applied to an endoprosthesis–radius construct; (**b**) endoprostheses inserted into PETG receptacles.

Torsion force was assessed due to the bone-anchorage type of the device. Its press-fit anchorage to the bone and screwing attachment between the PEEK part and threaded rod could be severely affected by natural torsional forces made by the dog. Two types of torsion tests were conducted with 30 specimens using a Handheld Digital Torque Gauge (BV.52295, SBV International Ltd., Brussels, Belgium) (Figure 6a).

In the first one, the torque was applied counterclockwise to the base of the PEEK part to find the loosening torque of five endoprostheses within their respective radii. In the second one, a clockwise torque was applied to the cap nut of 25 samples to find the point where the threaded rod began to rotate within the endoprosthesis; this force was named the rod loosening torque. For preparing the samples of the clockwise torque test, the endoprostheses were impacted inside a 3D-printed receptacle made of polyethylene terephthalate glycol-modified (PETG, Smart Materials, Jaen, Spain) (Figure 6b). Furthermore, biocompatible adhesives were applied on the rods to fix them inside the endoprostheses.

Butyl-cyanoacrylate adhesive was applied on threaded rods without a pre-treated surface to assess if the application area of this adhesive affected the rod loosening torque. This adhesive was applied throughout the threaded rod or at the bottom of the inner cavity of the PEEK part. Five samples were prepared for evaluating each application area.

Subsequently, three adhesives were used as threadlockers on threaded rods with surface treatment by cleaning with isopropanol, followed by sandblasting, and finishing with isopropanol to assess differences in rod loosening torque. These adhesives were butyl-cyanoacrylate adhesive (VetBond, 3M Co., Mineapolis, MN, USA), a diurethane dimethacrylate self-adhesive dual-cure resin cement in a paste (seT-PP, SDI Germany GmbH, Cologne, Germany), and a dental cement of zinc oxyphosphate (FORTEX, Faciden, Girona, Spain). Adhesives were applied in two ways. For the resin and zinc oxyphosphate cements, a thin layer was applied around the rod before inserting it into the endoprosthesis. For butyl-cyanoacrylate adhesive, two drops were deposited at the bottom of the inner cavity of the PEEK part before screwing the rod into it. Five samples were prepared for evaluating each adhesive.

#### 2.5.3. Fatigue Compression Test of the Endoprosthesis–Radius Construct

Dynamic tests were performed to study the long-term compression behavior. The fatigue load was determined by Equation (1) [35,54]. For a dog weight of 20 kg, considering the most unfavorable circumstances (dog at gallop), the maximum load to support would be approximately 400 N.

(1) Maximum load (N)=2 * DW (dog weight in kg)* 9.8

Fatigue testing was conducted under room conditions (22 ± 1 °C) using an electrodynamic testing machine with a load cell of 3 kN (ElectroPlus E3000, Instron, Norwood, MA, USA). Specimens were clamped perpendicular to the test table (Figure 5b) and tested in compression until failure or until a half million cycles, using a sinusoidal force cycle with a maximum load of 400 N and a load ratio of R = 0.1. A frequency of 2 Hz was used for the test, which is similar to the pace frequency when the dog is trotting. After fatigue testing, surviving specimens were quasi-static tested to determine the remaining strength as described in Section 2.5.2. Ten specimens were evaluated under these conditions.

### 2.6. Statistical Analysis

Compression and torque force with or without fatigue data were analyzed by analysis of variance (ANOVA) using the STATGRAPHICS program (XVII Centurion. Ver. 17.2.00, StatPoint, Inc., Herndon, VA, USA). When significant differences were observed (*p* ≤ 0.05), Fisher’s Least Significant Difference (LSD) was calculated.

## 3. Results

### 3.1. Mechanical Testing

#### 3.1.1. Mechanical Characterization of 3D-Printed PEEK Samples

The Poisson’s ratio value of the 3D-printed PEEK obtained in the present study ranged from 0.37 to 0.4. (Table 2). These results were similar to those of the canine radius and titanium alloys.

In addition, the Young’s modulus and tensile strength of the 3D-printed PEEK were closer to canine radius than metallic materials are. The Young’s modulus of the 3D-printed PEEK was 0.21-fold inferior to that of bone, while 316L-SS and Ti alloys exhibited a 17- and 6.96-fold higher modulus, respectively. Additionally, the tensile strength of the 3D-printed was 0.21-fold inferior to that of bone, while 316L-SS and Ti alloys exhibited a 2.79- and 3.38-fold higher tensile strength, respectively. However, the PEEK 3D-printed PEEK exhibited a reduction of tensile strength of 4.8- to 16.4-fold compared with the other materials. PEEK 3D-printed PEEK also exhibited a reduction of Young’s modulus by 4.7- to 80.6-fold compared with the other materials (SupplementaryMaterial_S1).

#### 3.1.2. Quasi-Static Vs Fatigue Compression Test of Endoprosthesis–Radius Construct

**Figure 7:**
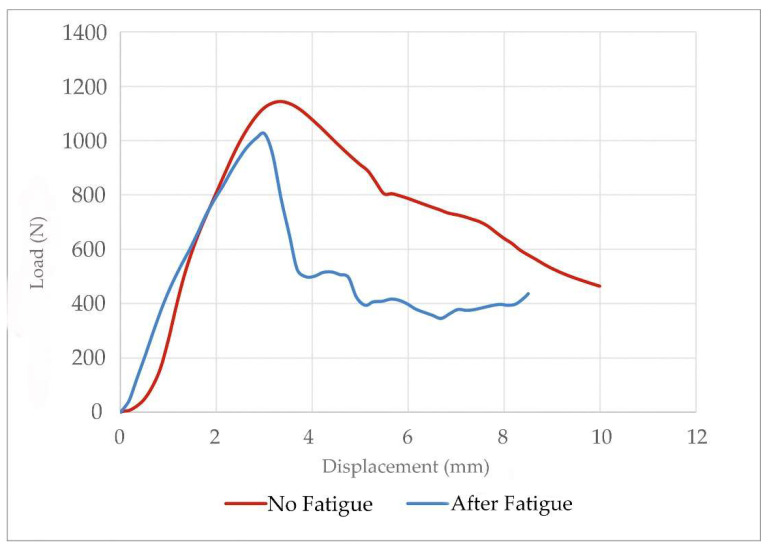
Compression force-displacement curve of an endoprosthesis–radius construct. Comparison of behavior before and after fatigue test.

None of the 10 tested constructs failed after applying the 500,000 cycles of fatigue. These fatigued constructs were subsequently assessed by quasistatic tests showing similar breaking loads to non-fatigued constructs (Table 3). In addition, the curves of the quasistatic tests were similar for fatigued and non-fatigued constructs (Figure 7). Before and after fatigue data showed that all constructs withstood more load than that calculated by Equation (1) (Table 3). The calculated FoS after fatigue was 2.70 ± 0.32 for the construct during galloping (Figure 5). The minimum result obtained in testing was two-fold above the calculated maximum load bearing by a 20 kg dog’s radius. The before and after fatigue force data for each sample can be found in the SupplementaryMaterial_S2.

The eight endoprosthesis–radius construct specimens assessed in the pure compression test showed two distinct types of failure: breakage of the PEEK part neck and threaded rod bending. In five cases, the endoprosthesis broke by the neck, making a displacement inside the radius medullary canal. In two of the specimens, the threaded rod flexed leading to the breaking of the bone. The failure mode of the specimens was similar for both construct types, before and after fatigue.

### 3.2. Torque Test of the Endoprosthesis–Radius Construct

**Figure 8:**
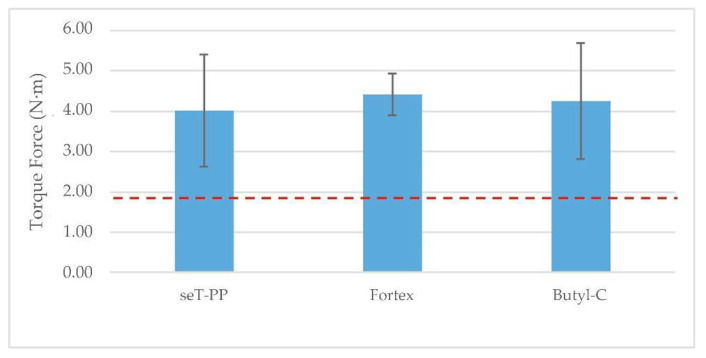
Data are the means with SD of the complete torque tests with force application at the threaded rod, with at least four replicates in each one. Significant differences are indicated with letters by LSD (*p* ≤ 0.05). The dashed line indicates the torque needed to spin round the endoprosthesis inside the medullary canal of the radius.

Three statistical comparisons were carried out (Table 4 and Table 5, Figure 8). Firstly, application areas of the butyl-cyanoacrylate on a non-treated threaded rod were compared and showed a significant difference between groups (Table 4). The butyl-cyanoacrylate adhesive application at the end of the threaded rod had the highest mean.

Secondly, the location of the torque force application on the endoprosthesis was evaluated (Table 5). A significantly higher mean was obtained for the application on the PEEK part instead of on the threaded rod. When the torque was applied on the threaded rod, the rod loosened. Meanwhile, if the torque was applied on the PEEK part, the whole endoprosthesis spun round and broke inside the medullary canal at the same time.

Finally, the type of adhesive applied on the pre-treated threaded rod was evaluated (Figure 8). No statistically significant differences were observed between these three adhesives. Zinc oxyphosphate cement showed the highest torque resistance with the lowest standard deviation of the three adhesives. However, all values were significantly higher than the torque needed to spin the endoprosthesis inside the medullary canal of the radius. The torsional force data for each sample group can be found in the SupplementaryMaterial_S3.

The tested samples showed three distinct types of failure: breakage between the endoprosthesis’ umbrella area and the distal area of the stem, the spinning of the threaded rod inside the endoprosthesis, and breakage of the threaded rod (Table 6). In all samples of the PEEK part tested and on one sample of the resin cement test, the endoprosthesis failed between the umbrella and the distal area of the stem—which ran into the distal end of the radius—while spinning inside the medullary canal. On one sample of the resin cement tests, three of the zinc oxyphosphate cement tests and three of the butyl-cyanoacrylate tests, the threaded rod spun inside the endoprosthesis, without any damage to the endoprosthesis after testing. Finally, on three samples of the resin cement tests, two of the zinc oxyphosphate cement tests and two of the butyl-cyanoacrylate tests, the threaded rod broke inside the endoprosthesis when reaching a torsional force of 5.00 N·m, without any damage to the endoprosthesis.

## 4. Discussion

The elliptical model of the endoprosthesis [45] is an appropriate option for weight-bearing of the canine radius. The construct bore higher compression forces than the canine radius during galloping [35]. All the compression results of our tests were more than twice the vertical load that the endoprosthesis would have to support during the canine’s life (400 N in the vertical position).

The compression tests showed a FoS of 2.70-fold the weight-bearing during galloping, even if the constructs were assessed at the fatigue test [35,55]. These satisfactory results were obtained using only wet canine radii without muscles or soft tissue of the forelimb. The variation between data were not statistically significant even when we could only standardize radii’ short diameters without finding a correlation between large and short diameters. Additionally, some radii were shorter than the average length for the same short diameter. These are positive outcomes because it is shown that anatomical differences affect the mode the bone bears at the force [46]. In these tests, both failure modes only affected the implant. In a clinical case, this would allow a substitution of the device while the bone remained intact.

Torque force resistance was assessed as this force is a mechanical limitation of the internal part of the exo-endoprosthesis. Preliminary tests of the interaction between the PEEK part and the threaded rod without adhesive were performed to evaluate if the adhesive solutions would contribute a significant improvement. The adhesives were selected because of their recognized biocompatibility, economic viability, easy application, and availability for a clinical situation [56].

Regardless of where the torque was applied, outcomes were low compared with the bone’s failure at the torque–radii fracture at slow torsion: 12.45 N·m [44]. A dog will almost never apply that torque value during a change of direction; in its normal biomechanics, lower values will always be reached. Thus, comparing the obtained results of the present study with bone torsional failure is not accurate. Even if an accident were to occur, the torsional force would preferably be absorbed by the medical device. This would protect the bone and reduce the possibilities of a complicated surgery. To the best of the authors’ knowledge, no studies were found concerning the natural dog’s torque force. For that reason, thorough studies concerning this topic should be carried out.

Application of the butyl-cyanoacrylate at the end of the threaded rod had a higher mean than around the rod. For a non-expert on adhesives, this result could be insightful because they might think that a greater quantity of adhesive would increase torque strength. The results could be explained by the cyanoacrylate adhesives’ behavior. Cyanoacrylates start the curing process in the presence of humidity [57]. When the butyl-cyanoacrylate is applied around the threaded rod, it cures completely before the threaded rod is totally inserted inside the PEEK part. The butyl-cyanoacrylate applied in thin layers shows a high-speed curing [57]. The thread’s shape is reduced and any chemical bonding between the threaded rod and PEEK part is broken. Meanwhile, if the butyl-cyanoacrylate is applied at the bottom of the part’s hole, it cures when the rod screws into the PEEK part spreading the adhesive, allowing it to tighten the threaded rod. In this case, the humidity is not enough to cure the whole volume of adhesive at the bottom of the PEEK part, and the butyl-cyanoacrylate adhesive just starts to cure when being spread as a thin layer along the rod during the screwing process.

In torsional tests, rod loosening occurred at presumably very low torque values; therefore, a treatment of the threaded rod before the application of an adhesive was considered, as Najeeb et al. [58] demonstrated in their study. The resistance to torque force observed after the treatment and with resin cement, zinc oxyphosphate cement, or butyl-cyanoacrylate was around two-fold that obtained if the force was applied on the PEEK part. This result is quite positive because if the exoprosthesis was attached to the PEEK part in a clinical case, and the PEEK broke, this would imply a more difficult surgery than if only the threaded rod loosened. Although no statically significant differences were obtained between these three adhesives, there were differences compared to the preliminary tests for butyl-cyanoacrylate. This difference could be related to the pre-treating made to the threaded rod on those three adhesives [58]. In spite of the statistical results, butyl-cyanoacrylate adhesive could be preliminary excluded as a permanent solution as it has been shown by its hydrolytic degradation in the body [59]. Considering other studies, resin cement could be the better option as a permanent adhesive above zinc oxyphosphate cement due to its greater lasting and better mechanical properties [56,60].

The Poisson’s ratio of the 3D-printed PEEK was similar to that of the canine radius and the titanium alloy. This means that the 3D-printed PEEK would contract and stretch when a force was applied, similar to the bone. Additionally, the 3D-printed PEEK had a closer Young’s modulus and elastic strength to the canine radius than the typical metal for orthopedic implants. Thus, only considering the comparison of the materials’ elastic properties, the 3D-printed PEEK could be an interesting candidate for substitution of metal materials to reduce the abovementioned stress-shielding phenomenon [26].

## 5. Conclusions

The evaluated endoprosthetic part of an exo-endoprosthesis for elliptical bone can greatly withstand a dog’s weight during a galloping pace and at a higher frequency than this pace without detriment to its maximum weight-bearing. This makes the 3D-printed PEEK exo-endoprosthesis a suitable mechanical choice for medical devices in veterinary medicine. However, more studies should be conducted in order to determine whether the threaded anchorage between the PEEK part and the rod would be strong enough to counteract a natural dog’s torque force during a change of direction at different paces.

## 6. Patents

Mendaza De Cal, R.M.; Peso Fernández, S.; Rodríguez Quirós, J. ES2736410. Endoprótesis a medida para huesos largos de animales 2020.

## Figures and Tables

**Table 1:** Characteristics of FFF printings on Simplify 3D slicer.

Number of models for each printing		2
Extrusion multiplier		0.92
Retraction distance (mm)		3.60
Retraction speed (mm/s)		30
Layer height (mm)		0.05
Top solid layers		9
Bottom solid layers		9
Perimeter shells		4
Skirt	Layer	1
	Skirt offset from part (mm)	0.00
	Skirt outlines	15
Infill (%)		50
Speed	Default (mm/s)	30
	Outline under speed (%)	50
	Solid infill under speed (%)	80
	Supports under speed (%)	80

**Table 2:** Mechanical properties of 3D-printed PEEK, canine radii, 316 stainless steel and titanium alloys.

Material	Tensile Strength (MPa)	Young’s Modulus (GPa)	Poisson’s Ratio
3D-printed PEEK (experimental)	36–52 [20]	2.1–2.9 [20]	0.37–0.4
Canine radius [48,49]	190.78–235.26	8.64–15.07	0.31–0.46
316L-SS [50,51,52]	90–1100	193–210	0.27
Ti alloys [52,53]	240–1200	88.5–155.9	0.33–0.35

**Table 3:** Fatigue effect on maximum force at failure of the construct. Results are shown as mean and standard deviation (SD).

	Maximum Force (N)
Quasi-static compression	1045.0 ± 78.0
Quasi-static compression after fatigue	1079.5 ± 40.1
MSE	30,333.2

Data are the mean of 8–10 replicates. Means were not significantly different by LSD. MSE = Mean Square Error.

**Table 4:** Mean and SD of groups to which the butyl-cyanoacrylate was applied on different areas of the threaded rod without surface pre-treatment.

Butyl-Cyanoacrylate	Maximum Torque (N·m)
Throughout threaded rod	0.30 ± 0.08 a
At the end of threaded rod	0.73 ± 0.14 b
MSE	0.06

Data are the mean of 4–5 replicates. Means were significantly different by LSD. MSE = Mean Square Error.

**Table 5:** Mean and SD deviation of force application area groups.

Application Area	Maximum Torque (N·m)
Threaded rod	0.73 ± 0.10 a
PEEK part	1.86 ± 0.09 b
MSE	0.04

Data are the mean of 4–5 replicates. Means were significantly different by LSD.

**Table 6:** Failure type at torque test versus sample groups.

Failure Type	Affected Sample Groups
Breakage of PEEK at umbrella–stem	PEEK part * Resin cement
Spinning of the threaded rod	Resin cement Zinc oxyphosphate cement Butyl-cyanoacrylate
Breakage of the threaded rod	Resin cement Zinc oxyphosphate cement Butyl-cyanoacrylate

* In this group, the torque was applied on the PEEK part not on the threaded rod.

## Data Availability

The original contributions presented in the study are included in the article/ datasets of the supplementary material.

## Supplementary Materials

The following supporting information can be downloaded at: https://www.mdpi.com/article/10.3390/ani12213021/s1, S1: SupplementaryMaterial_S1. Materials_ElasticProperties.xlsx; S2: SupplementaryMaterial_S2. Compression_with y without FAT.xlsx; S3: SupplementaryMaterial_S3. Torque tests.xlsx.
